# Supplementary material for: Visualizing Cell Death in Live Retina: Using Calpain Activity Detection as a Biomarker for Retinal Degeneration
Source: Int J Mol Sci. 2022 Mar 31;23(7):3892. doi: 10.3390/ijms23073892 (PMC8999672; doi:10.3390/ijms23073892)
Supplement: Supplementary file 1 [file ijms-23-03892-s001.zip › ijms-1603049-supplementary.pdf]

Supplementary Material: Visualizing cell death in live retina: Using calpain activity detection as a biomarker for retinal degeneration

Soumaya Belhadj, Nina Sofia Hermann, Yu Zhu, Gustav Christensen, Torsten Strasser, François Paquet-Durand

Table of Contents

Figure S1
Figure S2

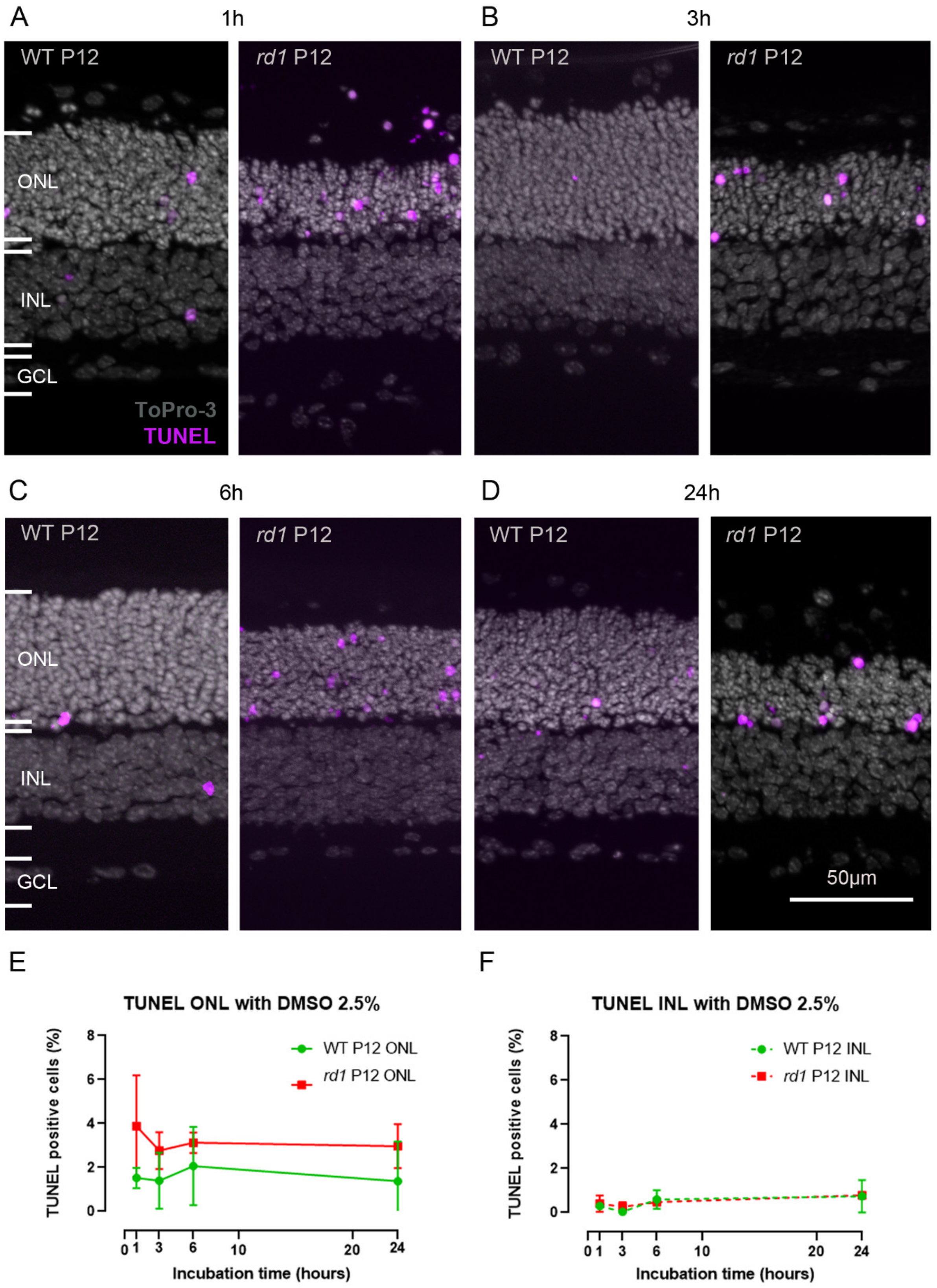

Figure S1. Cell death in live tissue treated with 2.5 % DMSO (vehicle). The TUNEL assay (magenta) for cell death detection was carried out on fixed retinal sections, To-Pro-3 (grey) was used as nuclear counterstaining. Organotypic retinal explant cultures derived from the *rd1* mouse model were incubated with: (**A**) DMSO (2.5 %) for 1 h, (**B**) 3h, (**C**) 6 h, (**D**) and 24 h. (**E**, **F**) Percentages of TUNEL positive cells in the outer nuclear layer (ONL) and inner nuclear layer (INL). Images are representative for results obtained from at least 3 independent retinal

explant cultures; error bars indicate STD; statistical analysis: two-way ANOVA with Holm-Sidak's multiple comparisons test, GCL = ganglion cell layer.

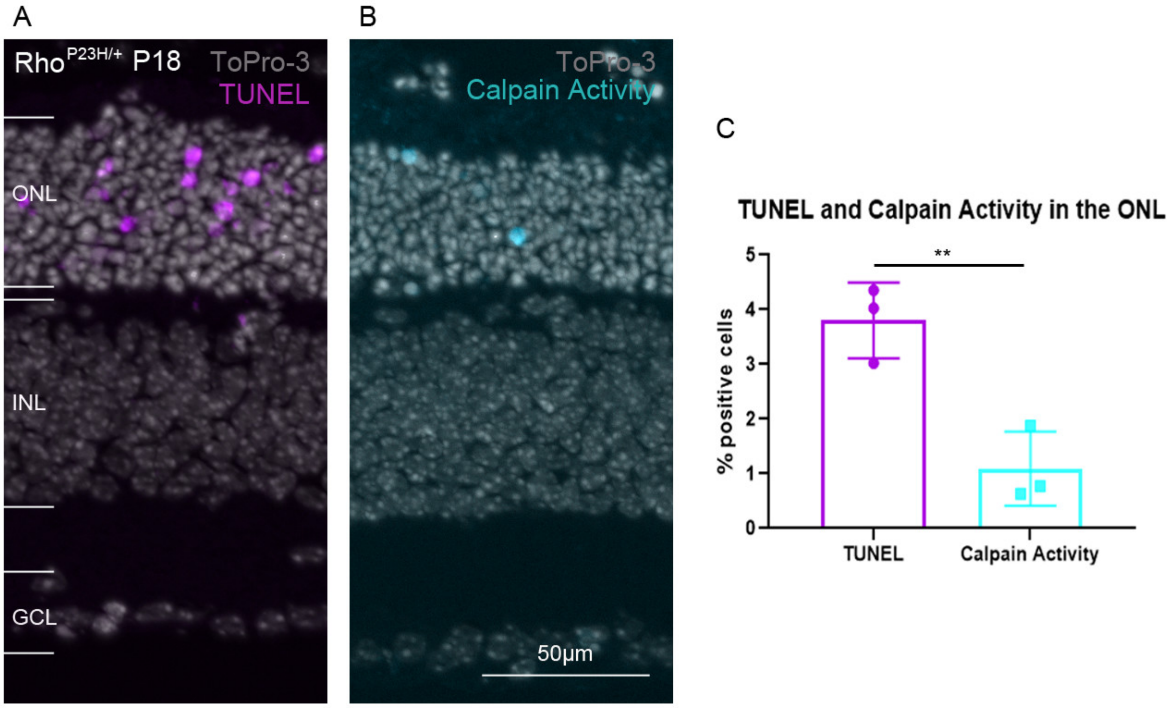

Figure S2. Live tissue detection of calpain activity and cell death in the *Rho*$^{P23H/+}$ mouse model. (**A**) The TUNEL assay (magenta) for cell death detection was carried out on fixed tissue sections from organotypic retinal explant cultures derived from the *Rho*$^{P23H/+}$ mouse model. (**B**) Live, organotypic retinal explant cultures were incubated with 50 µM CMAC for 6 h at P18. A calpain specific fluorescent signal (cyan) was observed in individual photoreceptor cells in the outer nuclear layer (ONL). To-Pro-3 (grey) was used as nuclear counterstaining. Images representative for results obtained from at least 3 independent retinal explant cultures; error bars indicate STD; statistical analysis: unpaired t-test. INL = inner nuclear layer, GCL = ganglion cell layer.
